# Effect of freeze-thaw cycling on grain size of biochar

**DOI:** 10.1371/journal.pone.0191246

**Published:** 2018-01-12

**Authors:** Zuolin Liu, Brandon Dugan, Caroline A. Masiello, Leila M. Wahab, Helge M. Gonnermann, Jeffrey A. Nittrouer

**Affiliations:** 1 Department of Earth, Environmental, and Planetary Sciences, Rice University, Houston, Texas, United States of America; 2 Departments of Chemistry and Bioscience, Rice University, Houston, Texas, United States of America; RMIT University, AUSTRALIA

## Abstract

Biochar may improve soil hydrology by altering soil porosity, density, hydraulic conductivity, and water-holding capacity. These properties are associated with the grain size distributions of both soil and biochar, and therefore may change as biochar weathers. Here we report how freeze-thaw (F-T) cycling impacts the grain size of pine, mesquite, miscanthus, and sewage waste biochars under two drainage conditions: undrained (all biochars) and a gravity-drained experiment (mesquite biochar only). In the undrained experiment plant biochars showed a decrease in median grain size and a change in grain-size distribution consistent with the flaking off of thin layers from the biochar surface. Biochar grain size distribution changed from unimodal to bimodal, with lower peaks and wider distributions. For plant biochars the median grain size decreased by up to 45.8% and the grain aspect ratio increased by up to 22.4% after 20 F-T cycles. F-T cycling did not change the grain size or aspect ratio of sewage waste biochar. We also observed changes in the skeletal density of biochars (maximum increase of 1.3%), envelope density (maximum decrease of 12.2%), and intraporosity (porosity inside particles, maximum increase of 3.2%). In the drained experiment, mesquite biochar exhibited a decrease of median grain size (up to 4.2%) and no change of aspect ratio after 10 F-T cycles. We also document a positive relationship between grain size decrease and initial water content, suggesting that, biochar properties that increase water content, like high intraporosity and pore connectivity large intrapores, and hydrophilicity, combined with undrained conditions and frequent F-T cycles may increase biochar breakdown. The observed changes in biochar particle size and shape can be expected to alter hydrologic properties, and thus may impact both plant growth and the hydrologic cycle.

## Introduction

Biochar has been proposed as a means to sequester carbon and to improve soil properties over the long term [1–3]. To accomplish these goals simultaneously, biochar must have a long residence time in soil and it must maintain the ability to offer positive ecosystem services (e.g. improved nutrient retention, improved soil water properties) while in soil [4, 5]. Previous studies have characterized the physical and chemical properties of freshly produced biochar that offer ecosystem services (e.g. grain size, porosity, ion exchange capacity, sorption capacity) [6–8], however, little is known about how these properties evolve over time. For example, frequent freeze and thaw cycling (F-T) in cold regions [9], penetration by plant roots or fungal hyphae [10], decomposition, and bioturbation [11] all likely reduce biochar grain size, although the timescales of these processes are not well constrained.

It is important to understand how freeze-thaw cycling alters biochar grain size and porosity because these parameters have been shown to impact soil hydrologic properties such as hydraulic conductivity [4, 12, 13] and soil water retention [14, 15]. Existing research suggests that whether biochar increases or decreases soil hydraulic conductivity is a function of the difference in grain size between biochar particles and soil particles [12]. When fine biochar grains infill pores between coarse soil grains (e.g. sand), this increases tortuosity, reduces interpore size and pore throat size, and thus decreases hydraulic conductivity [8, 12, 16]. When biochar is coarser than soil grains (e.g. clay), pore sizes increase, resulting in an increase of hydraulic conductivity [16, 17]. Any biochar particle size changes resulting from freeze-thaw cycles may therefore drive changes in soil hydraulic conductivity.

The internal porosity of biochar particles (intraporosity) also plays a role in delivering ecosystem services. The internal pore volume of biochar from plant matter is dominated by large pores remaining from the plant cell skeleton [18]. These large pores play a key role in increasing plant-available water in biochar-amended soils, as demonstrated water retention curves of biochar-sand mixtures where biochar particle size varied [14]. Liu et al. [14] documented that coarse biochar caused a larger increase in plant-available water than fine biochar, pointing to a key role for these large, internal pores in holding soil water in a plant-available form.

Biochar’s intraporosity (ϕ_intra_, m^3^/m^3^) is high, up to 0.86 m^3^/m^3^ [18]. When water inside these pores expands during freezing, grains may break into finer grains, and we hypothesize that biochar grain size will be reduced by this effect. To test this hypothesis, we investigated the grain size response of different types of biochar, at different water contents, to F-T cycling. We evaluated four types of biochar (pine, mesquite, miscanthus, and sewage waste) pyrolyzed at 400°C. We conducted 1, 2, 5, 10, and 20 F-T cycles on these four types of biochars after they were placed in a synthetic rainwater bath for at least one day and sealed in a cylinder without drainage. We measured the grain-size distribution of the biochar, skeletal density (ρ_s_, kg/m^3^, the density of the biochar skeleton not considering internal pores) and envelope density (ρ_e_, kg/m^3^, the density of biochar particles including internal pores) pre- and post- F-T cycles. We then calculated the intraporosity (ϕ_intra_ = 1- ρ_e_/ρ_s_, m^3^/m^3^) of biochar particles to quantify any changes in intrapores. Changes in skeletal density or envelope density will yield changes of intraporosity. However, even if there is no statistical change for skeletal density or envelope density, we cannot definitively conclude that there is no change of intraporosity without making strong assumptions (e.g. independence of skeletal density and envelope density). Using our observations, we developed a conceptual model of how the grain size of biochar is changed by F-T cycles when samples were prepared without drainage. Based on these results, we then tested how F-T cycles affect the grain size distribution of mesquite biochar after 30 minutes of gravity-driven drainage.

## Materials and methods

### Synthetic rainwater

We prepared synthetic rainwater by dissolving NaCl, KNO_3_, MgSO_4_, CaSO_4_, and NH_4_NO_3_ into purified water (18.2 MΩ-cm, PURELAB® Ultra Laboratory Water Purification Systems, SIEMENS, Germany) (S1 Table). This recipe represents an average for U.S. inland rainwater [19].

### Biochar production

**Fig 1:**
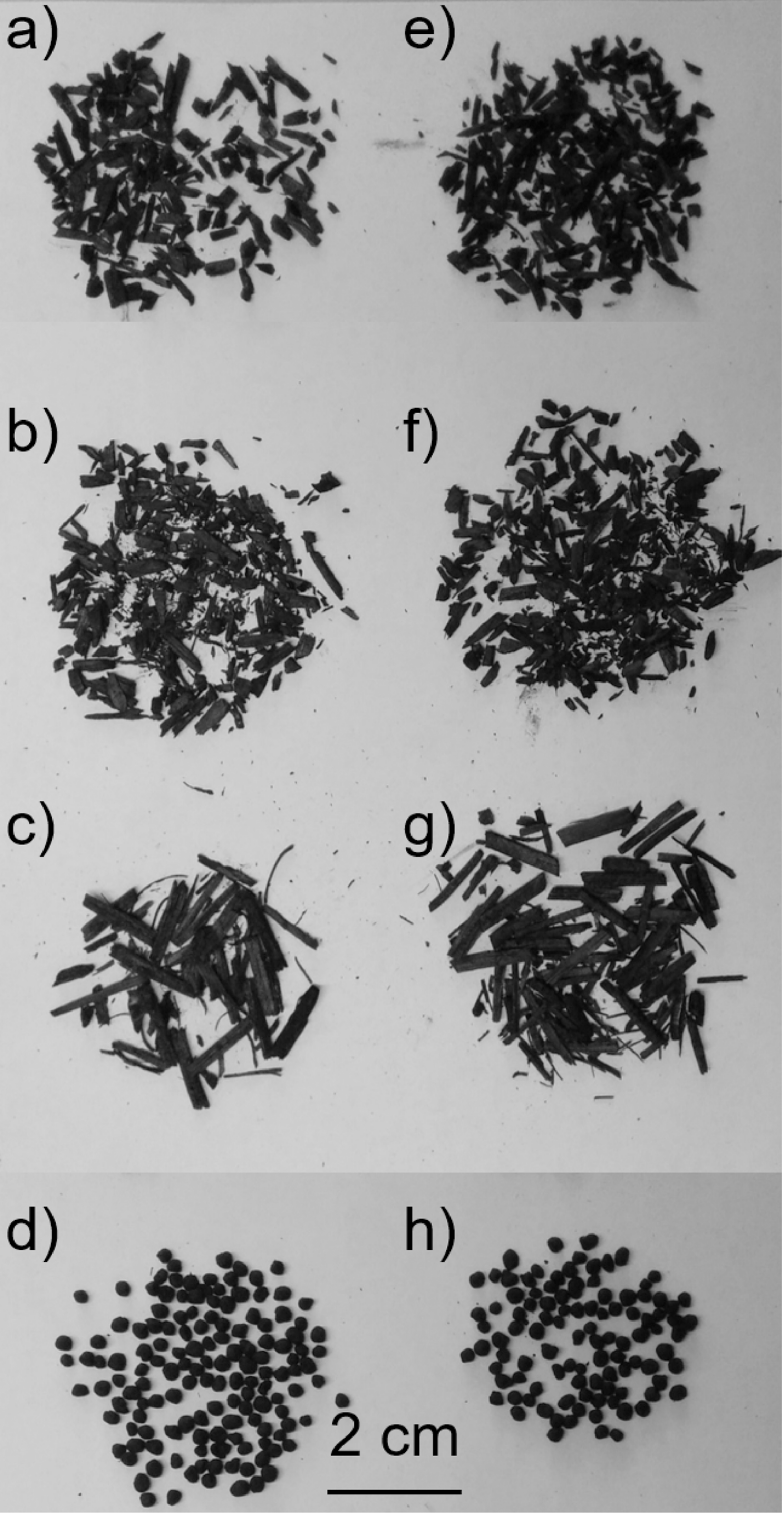
**Photograph of (a) pine biochar; (b) mesquite biochar; (c) miscanthus biochar; (d) sewage waste biochar pre F-T cycling; and (e) pine biochar; (f) mesquite biochar; and (g) miscanthus biochar; (h) sewage waste biochar after 20 F-T cycles.** These biochars were placed in a water bath for at least one day and sealed in cylinders without drainage pre F-T cycling. The images show that biochars made from different feedstocks are visually different. However, for the same biochar type there was no visual difference between pre- and post- F-T cycles. Biochar feedstocks were sieved between 2.36–3.35 mm.

We pyrolyzed pine, mesquite, miscanthus, and pelletized sewage waste feedstock at 400°C for 4 hrs to form biochar (Fig 1) [12, 20]. To minimize heat transfer differences caused by different grain sizes [21], we pre-ground pine, mesquite, and miscanthus feedstocks and sieved the four feedstocks into a uniform size range (2.36–3.35 mm, corresponding to #8 to #6 US standard mesh) prior to pyrolysis. During pyrolysis, the heating rate was 5°C/min until the furnace reached 400°C, and then temperature was maintained at 400°C for 4 hrs. The biochar then cooled down in the absence of oxygen for a minimum of 16 hrs. For each type of biochar, we made several batches, homogenized them, and stored them in sealed glass jars until use in the experiments. We made four batches of pine biochar, seven batches of mesquite biochar, five bathes of miscanthus biochar, and one batch of sewage waste biochar.For biochar production, we used 149 ± 7 g pine, 196 ± 10 g mesquite, 101 ± 1 g miscanthus, and 550 ± 0 g sewage waste feedstock; the biochar mass yields were 54 ± 4 g, 80 ± 4 g, 36 ± 2g or 297 ± 0 g, respectively. See statistical analysis for details on values and errors.

### Freeze-thaw experiments

For each freeze-thaw experimental sample, we poured 10 g of air-dried biochar into a clear, plastic, cylindrical column (0.0198 m inner radius) with a piece of 54-micron polyester mesh (Part No.: CMY-0054-10YD, Small parts, FL) on the bottom of the column to allow water flow while preventing biochar mass loss. We placed the base of columns into a synthetic rainwater bath to allow water to rise into the sample from bottom to top. The water level was at least 5 cm higher than the sample height. However, according to visual inspection, pine, mesquite, and miscanthus biochars initially floated when we added water and approximately half of the biochar sank overnight. The majority of the sewage waste biochar sank after being placed in water as its envelope density is greater than the density of water. While not perfectly saturated, our experiment may emulate saturation after rainfall.

The presence of salts on the surface of biochar particles [22] may affect water infiltration into particles as the salts dissolve and pores may become open for water flow. This would increase settling of particles in the water column as pores are filled with water. However, our previous study [12] documented an ash content of approximately 5% for mesquite biochar made by the same techniques suggesting low content of inorganic elements and thus low potential for inorganic salts. Therefore, we assume salt effects are negligible in our experiments. After allowing each sample to remain in the rainwater bath for at least one day, we prepared samples with two drainage conditions which produced different initial water contents (Table 1): (1) undrained for all biochars (referred as the ‘undrained experiment’) resulting in samples with water contents that ranged from 1.3 ± 0.5 to 11 ± 3 kg/kg; and (2) 30 minutes of gravity-driven drainage for mesquite biochar only (referred as the ‘drained experiment’) resulting samples with water contents of 1.9 ±0.3 kg/kg. We covered the bottom and the top of each column with plastic wrap and then sealed the whole column in a freezer zip-lock bag to minimize water loss.

**Table 1:** Basic information of the undrained and drained experiments in this study.

Type	Information
*Undrained experiment*	
Biochar type	Pine, mesquite, miscanthus, and sewage waste
Freeze-thaw cycles	1, 2, 5, 10 and 20
Measurements	Grain size, skeletal density, envelope density and intraporosity
*Drained experiment*	
Biochar type	Mesquite
Freeze-thaw cycles	1, 2, 5 and 10
Measurements	Grain size

We froze biochars at -19 ± 3°C for 8 hrs in a freezer, and thawed them at room temperature (25 ± 0°C) for 16 hrs for each F-T cycle. In the undrained experiment, we completed three replicates of four types of biochar treated by 1, 2, 5, 10, and 20 F-T cycles, for a total of 60 samples. In the drained experiment, we made three replicate measurements of mesquite biochar treated by 1, 2, 5, and 10 F-T cycles, for a total of 12 samples. Between F-T cycles, we monitored total mass of each sample after thawing and before freezing and added synthetic rainwater to the top of the sample to keep the water content constant through F-T cycling by assuming no biochar mass loss. We also monitored the temperature of the freezer, the room, and three representative biochar samples (1 mesquite, 1 miscanthus, and 1 sewage waste) to ensure complete freezing (S1 Text and S1–S3 Figs).

After a set number of F-T cycles (e.g., 1, 2, 5, 10, or 20), we transferred each sample into a glass jar and massed the wet sample (M, kg). After measuring the wet mass, we oven-dried each sample at 60°C for 72 hrs in open glass jars. After drying we sealed the air-tight jars to avoid water adsorption during exposure to air and measured the mass of each dry sample (M_d_, kg). We then calculated water content (*θ = M*/*M*_*d*_-1, kg/kg). We also computed mass loss ((1-*M*_*d*_/*M*_*o*_) x 100, %) using sample mass pre F-T (*M*_*o*_) and *M*_*d*_. The mass losses were 4 ± 4%, 4 ± 1%, 6 ± 3%, 3 ± 3% for pine, mesquite, miscanthus, and sewage waste biochar, respectively. Most of the mass losses were due to handling, and had no effect on further calculations.

### Grain size analysis

We used a Camsizer (Retsch Technology, Germany) to make quantitative grain size and grain shape measurements. The Camsizer can measure particle size ranges from 0.02 to 30 mm with a maximum inaccuracy of 0.1 micron per object. Digital cameras within this instrument take 60 images s^-1^ as particles fall freely through air and the instrument then calculates a range of parameters that characterize particle shape and size. We report grain size properties of biochars including grain diameter at the shortest chord (D_min_), and grain size at the maximum diameter (D_max_) of a biochar grain projection pre- and post- F-T cycles (S4 Fig). From the grain-size distribution, we determined median grain size (D_min50_ and D_max50_) and calculated the aspect ratio (A_R_ = D_min50_ / D_max50_). For D_min_ and D_max_ we report mean and standard deviation of triplicate measurements. For A_R_, mean values are calculated as well as errors (δ_AR_),

$$\delta_{AR}=A_{R}\sqrt{{\left(\frac{\delta_{D\min}}{D_{\min}}\right)}^{2}+{\left(\frac{\delta_{D\max}}{D_{\max}}\right)}^{2}}$$ (1)

where δ_Dmin50_ and δ_Dmax50_ are the standard deviations of D_min50_ and D_max50_, respectively.

We performed a secondary grain-size analysis on the fine and coarse fractions of the biochar to determine if the coarse and fine fractions had similar grain shapes. We sieved pine, mesquite, and miscanthus biochars after 20 F-T cycles using a #20 U.S. standard mesh resulting in particles smaller than 0.853 mm (referred as ‘fine’) and larger than 0.853 mm (referred as ‘coarse’). We then measured the grain-size distribution of the fine and the coarse fractions of the biochar and calculated their A_R_ values.

### Modified hyperbolic model

We used a hyperbolic model to provide a continuous grain-size distribution from discrete grain size observation of the biochar as Bayat et al. [23] showed that hyperbolic model has the highest fitting accuracy over a wide range of grain size and soil textures. The hyperbolic model [24] correlates the percent of grains by volume (P) passing through the a specific diameter (D) through two empirical constants (A, c).

$$P=50\left(1+\tanh\left(\frac{D-A}{c}\right)\right)$$ (2)

To fit the bimodal grain size distribution for our biochar, we modified the hyperbolic model to account for two grain size distribution curves (Eq 3).

$$P=50\sum_{i=1}^{2}w_{i}\left(1+\mathrm{Tanh}\left(\frac{D-A_{i}}{c_{i}}\right)\right)$$ (3)

Where w_i_ is the weight for each grain size distribution curve and w_1_+w_2_ = 1. We can only fit w_1_ because w_2_ = 1- w_1_.

We used this modified hyperbolic model to fit D_max_ and D_min_ distribution at 0 and 20 F-T cycles in the undrained experiment. The fitted parameters (A, c, and w_1_) and goodness of fit (R-square (R^2^) and root-mean-square error (RMSE)) were determined using the MATLAB Curve Fitting toolbox.

### Skeletal density, envelope density, and intraporosity

We characterized how skeletal density, envelope density, and intraporoisty changed as the grain size changed due to F-T cycles. We measured the ρ_s_ of biochar in the undrained experiment by helium pycnometry in a 3.5 cc sample chamber (AccuPyc II 1340, Micromeritics, Norcross, GA) and the ρ_e_ of biochar using quasi-fluid displacement in a sample chamber with inside diameter of 1.27 cm (Geopyc 1360, Micromeritics, Norcross, GA). Both the AccuPyc and Geopyc measure density by measuring the displacement of a fluid. In the case of the AccuPyc, the fluid is He atoms. In the case of the Geopyc, the “fluid” is composed of Dry Flo particles, a quasi-fluid composed of small, rigid spheres having a high degree of flow-ability and a particle diameter of ~120 μm.

For skeletal density measurements, the approximate sample masses were 0.7g, 0.8g, 0.4g, and 1.5g for pine, mesquite, miscanthus, and sewage waste biochar, respectively. When measuring envelope density of pine, mesquite, miscanthus, and sewage waste biochar, we used approximately 0.3g, 0.4g, 0.2g, and 1g sample, respectively. For details on measurement of ρ_s_ and ρ_e_, see Brewer et al. [18]. We then calculated intraporosity of biochar grains (ϕ_intra_ = 1-ρ_e_/ρ_s_, m^3^/m^3^). We report mean and standard deviation of triplicate measurements for ρ_s_ and ρ_e_. For ϕ_intra_, we report mean values and error (δ _ϕintra_) was calculated as,

$$\delta_{\phi \mathrm{int}ra}=\phi_{\mathrm{int}ra}\sqrt{{\left(\frac{\delta_{\rho s}}{\rho_{s}}\right)}^{2}+{\left(\frac{\delta_{\rho e}}{\rho_{e}}\right)}^{2}}$$ (4)

where δ_ρs_ and δ_ρe_ are the standard deviations of ρ_s_ and ρ_e_, respectively.

The Geopyc provides the best envelope density data when the displacement fluid particles (diameter ~120 μm) are at least 20x smaller than the particles being measured. This allows the displacement fluid to completely surround the measured particles. In practice this restricts envelope density measurements to particles with a diameter larger than 2 mm. Before F-T cycling, a small fraction (D_max50_ were 1 ± 0.4%, 1.3 ± 0.4%, 2.0 ± 0.6%, 0.6 ± 0.5% by volume for pine, mesquite, miscanthus and sewage waste biochar, respectively) of biochar particles were finer than 2 mm. After F-T cycling, the volumetric fraction of fine particles (D_max50_ < 2 mm) increased to 19 ± 2%, 23 ± 3%,29 ± 2% and 1.7 ± 0.6% for pine, mesquite, miscanthus and sewage waste biochar, respectively. The existence of these fine particles causes inaccuracy of envelope density measurements by the Geopyc and thus changes in envelope density caused by F-T cycling may not have be detected. However, we still report envelope density data to provide basic information of the biochars used.

### Statistical analysis

Statistical comparisons of D_min50_, D_max50,_ A_R_, ρ_s_, ρ_e_, and ϕ_intra_ between pre- and post- F-T cycles, and between different biochars were done using one-way analysis of variance (ANOVA). Differences were deemed significant at a *p*-value less than 0.05. We also computed Pearson correlation coefficients (R) between the decrease of median grain size and water content of samples at 10 F-T cycles (the most F-T cycles in drained experiment).

For each experiment, we had triplicate samples that we analyzed. All values and errors presented here are mean and standard deviation for direct measurements. For ϕ_intra_ and A_R_, values are mean and errors are calculated using equations Eq 1 or Eq 4, respectively.

## Results

### Grain size and aspect ratio, undrained experiment

**Fig 2:**
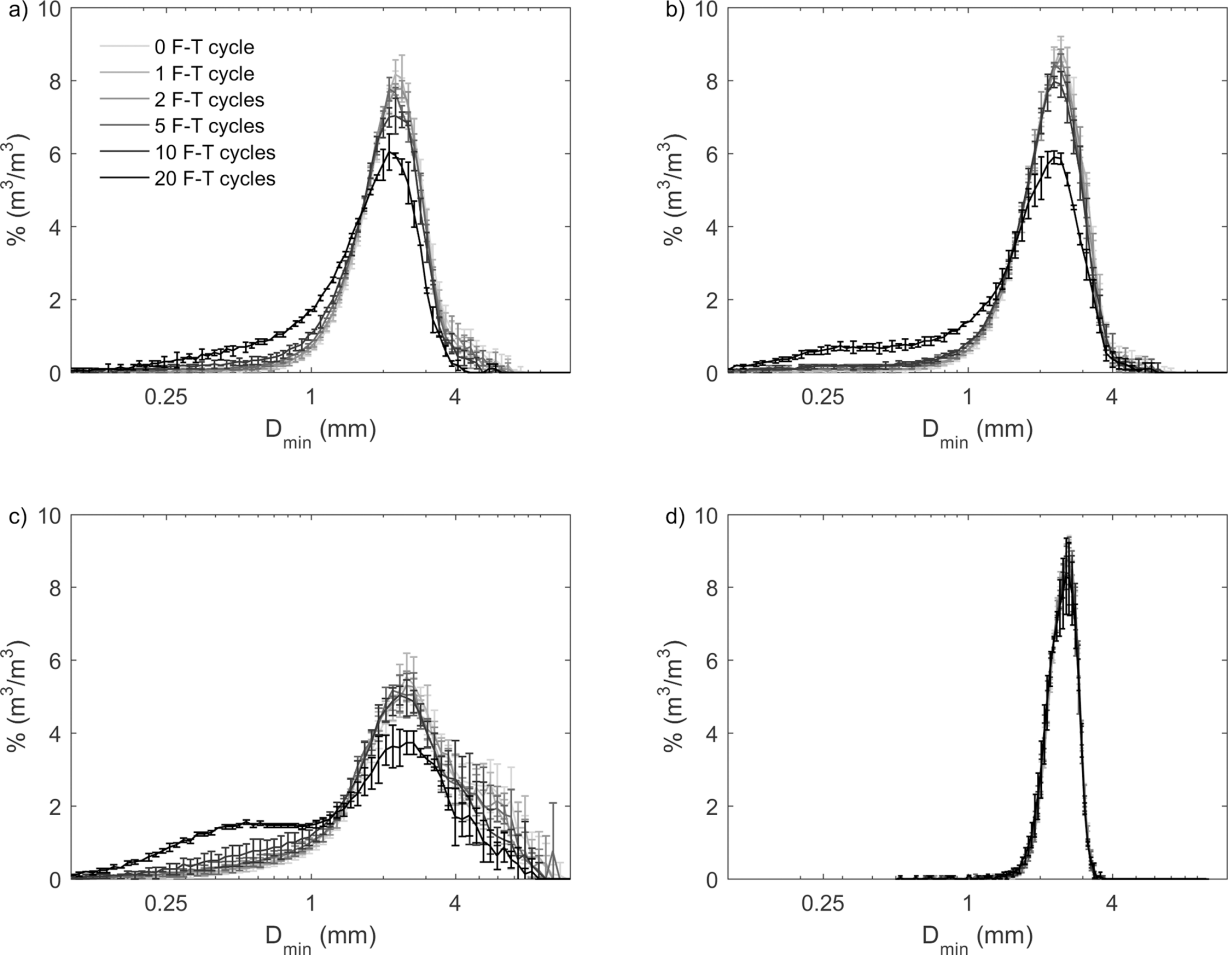
**Grain size (at the shortest chord of a biochar grain projection, D**_**min**_**) distribution by volume for (a) pine; (b) mesquite; (c) miscanthus; and (d) sewage waste biochar from 0–20 F-T cycles in the undrained experiments.** There were decreases in D_min_ for pine, mesquite, and miscanthus biochars with increasing number of F-T cycles. However, distributions of D_min_ for sewage waste biochar from 0–20 F-T cycles overlapped with each other, meaning that there is no change of D_min_ distribution for sewage waste biochar. We didn’t observe any particle aggregation. Values and errors are mean and standard deviation of triplicate samples. Numerical data for individual samples are presented in S2 Table.

**Fig 3:**
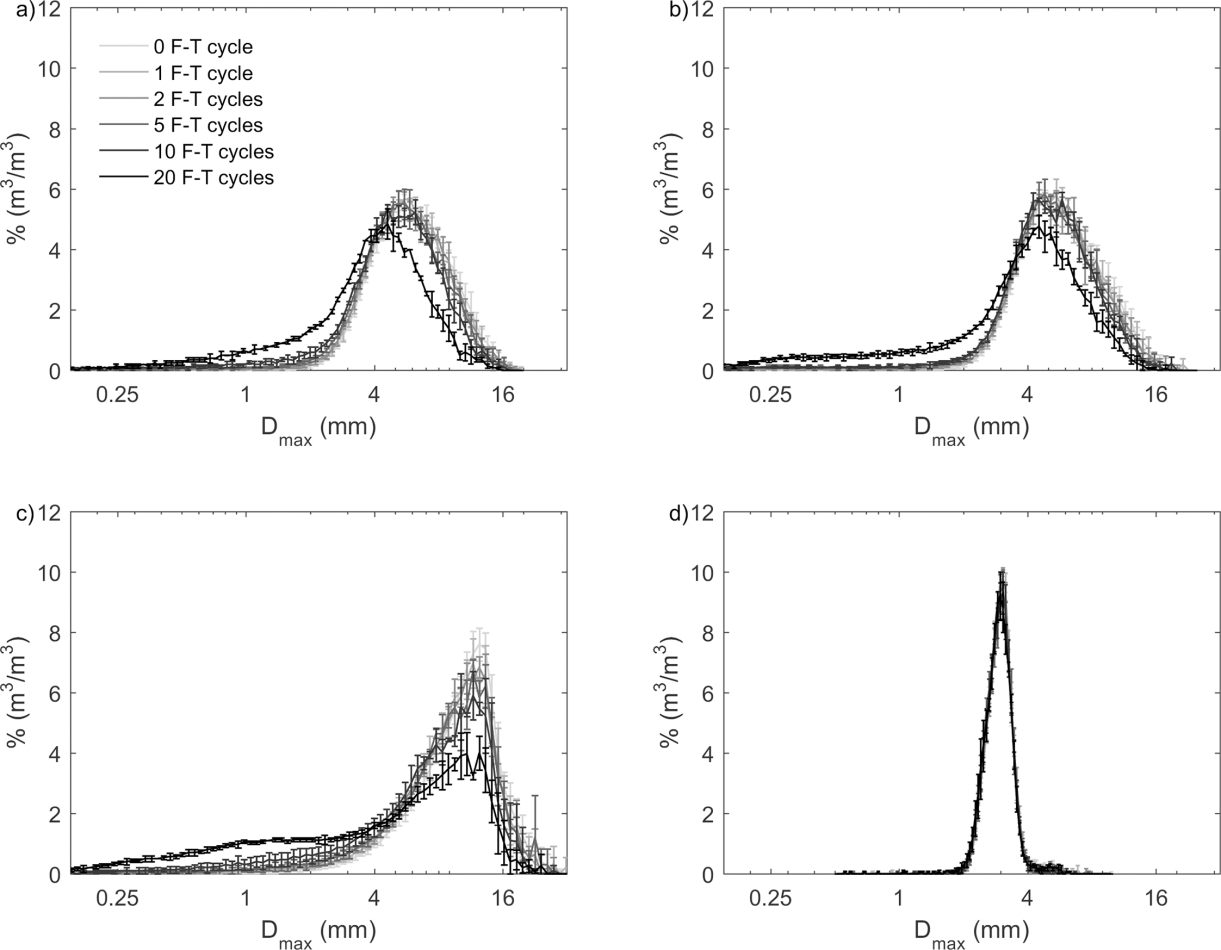
**Grain size distribution (at the maximum diameter of a biochar grain projection, D**_**max**_**) by volume for (a) pine; (b) mesquite; (c) miscanthus; and (d) sewage waste biochar from 0–20 F-T cycles in the undrained experiments.** There were decreases in D_max_ for pine, mesquite, and miscanthus biochars with increasing number of F-T cycles. However, distributions of D_max_ for sewage waste biochar from 0–20 F-T cycles overlapped with each other, meaning that there is no change of D_max_ distribution for sewage waste biochar. Values and errors are mean and standard deviation of triplicate samples. Numerical data for individual replicates are presented in S2 Table.

In the undrained experiment, all biochars had unimodal grain-size distributions before F-T cycling (Figs 2 and 3). Pine biochar and mesquite biochars had similar D_min_ and D_max_ distributions. The grain-size distribution of miscanthus biochar was wider than that of pine and mesquite biochars. The grain-size distribution of sewage waste biochar was narrower than that of pine, mesquite, and miscanthus biochars.

**Fig 4:**
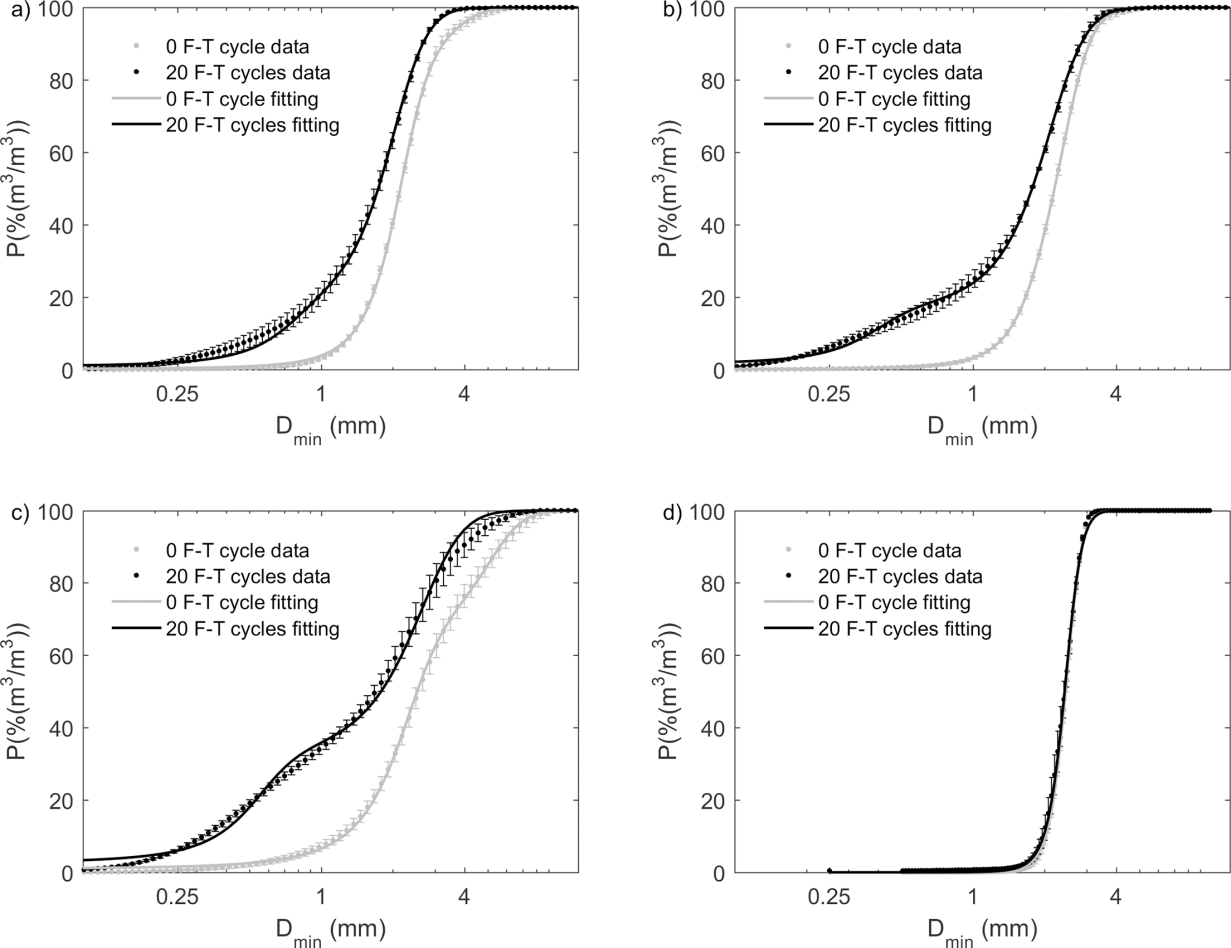
**Cumulative grain size (D**_**min**_**) distribution by volume at 0 and 20 F-T cycles and the best-fit modified hyperbolic model for (a) pine; (b) mesquite; (c) miscanthus; and (d) sewage waste biochar in the undrained experiment.** Values and errors are mean and standard deviation of triplicate samples. Numerical data for individual replicates are presented in S2 Table.

**Fig 5:**
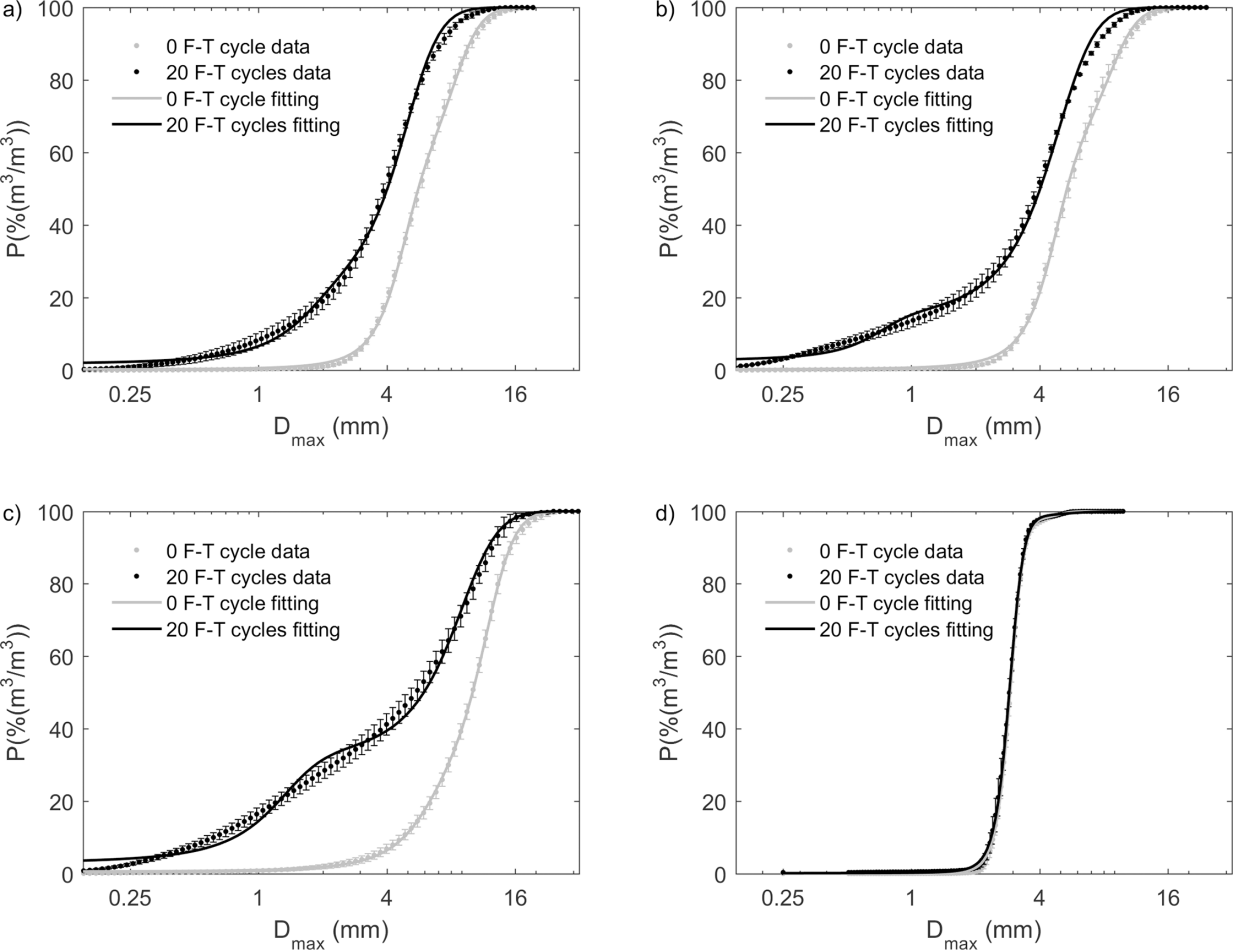
**Cumulative grain size (D**_**max**_**) distribution by volume at 0 and 20 F-T cycles and the best-fit modified hyperbolic model for (a) pine; (b) mesquite; (c) miscanthus; and (d) sewage waste biochar in the undrained experiment.** Values and errors are mean and standard deviation of triplicate samples. Numerical data for individual replicates are presented in S2 Table.

Modified hyperbolic model fitting parameters were very similar between pine biochar and mesquite biochar. We observed gaps of cumulative grain size distribution indicating decreases in grain size between 0 and 20 F-T cycles, and the decreases were quantified by the change of modified hyperbolic model fitting parameters for pine, mesquite and miscanthus biochar. However, for sewage waste biochar, cumulative grain size distribution at 0 F-T cycle overlapped with that at 20 F-T cycles and the hyperbolic model fitting parameters at 0 F-T cycle were also very close to that at 20 F-T cycles. This indicates no change in grain size for sewage waste biochar. In addition, the weight parameter (w_1_) for sewage waste is 1 which means w_2_ is equal to 0 indicating a unimodal grain size distribution (Figs 4 and 5 and Table 2).

**Table 2:** Modified hyperbolic model parameters and goodness of fit include: The weighting factors (w_1_), constants A and c for two sub grain size distribution curves, R-square (*R*^*2*^) and root-mean-square error (*RMSE*).

Biochar	# of F-T cycle	w_1_	A_1_	A_2_	c_1_	c_2_	R^2^	RMSE (%(m^3^/m^3^))
				*D*_*min*_				
Pine	0	0.88	2.1	3.6	0.70	1.2	1.000	0.46
	20	0.86	1.9	0.7	0.80	0.30	1.000	0.74
Mesquite	0	0.73	2.4	1.8	0.75	0.56	1.000	0.19
	20	0.86	1.9	0.36	0.91	0.18	1.000	0.86
Miscanthus	0	0.68	2.1	4.9	0.97	1.9	1.000	0.75
	20	0.72	2.3	0.50	1.3	0.24	0.998	1.9
Sewage waste	0	1.0	2.4	0.0	0.39	0.0	1.000	0.63
	20	1.0	2.4	0.12	0.41	0.41	1.000	0.66
				*D*_*max*_				
Pine	0	0.52	4.6	7.9	1.4	2.8	1.000	0.64
	20	0.83	4.5	1.6	2.2	0.82	0.998	1.60
Mesquite	0	0.58	4.5	8.1	1.4	3.0	1.000	0.69
	20	0.89	4.3	0.66	2.4	0.3	0.998	1.80
Miscanthus	0	0.81	11	5.7	4.2	2.4	1.000	0.44
	20	0.71	7.9	1.1	4.4	0.7	0.997	2.21
Sewage waste	0	0.98	2.9	4.9	0.44	0.7	1.000	0.42
	20	0.97	2.9	3.9	0.45	2.6	1.000	0.43

**Fig 6:**
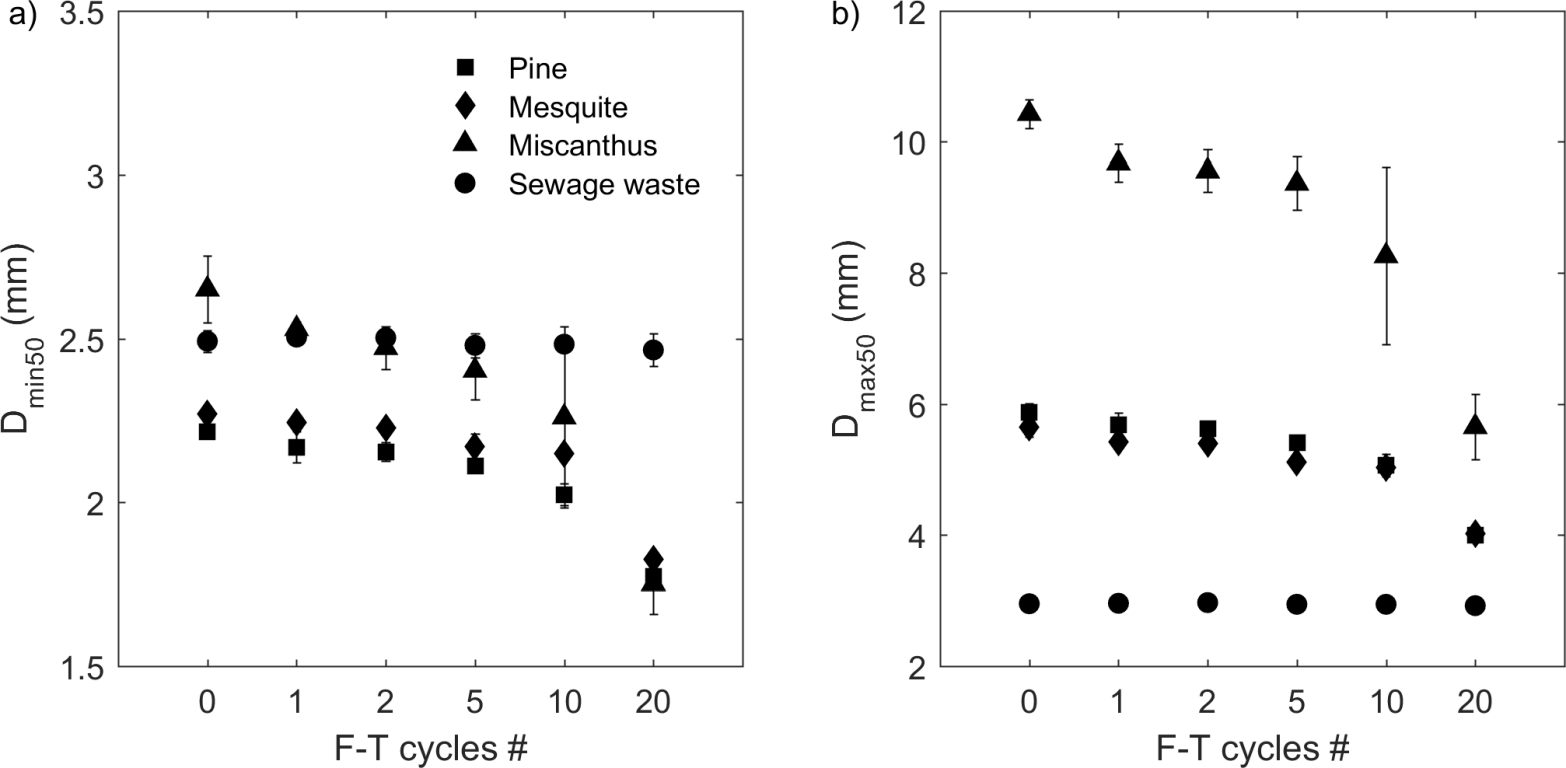
**Median grain size (a) at the shortest chord (D**_**min50**_**) and (b) at the maximum diameter (D**_**max50**_**) of all undrained experiment biochar samples from 0 to 20 F-T cycles.** Note: X-axis is not on a linear scale. Values and errors are mean and standard deviation of triplicate samples. Numerical data for individual replicates are presented in S2 Table.

Before F-T cycling, the median grain size varied with biochar feedstocks even though they were sieved to the same mesh sizes pre-pyrolysis (Fig 6). Pine and mesquite biochars had similar but statistically different D_min50_ (2.2 ± 0.0 mm and 2.3± 0.0 mm for pine and mesquite biochars, respectively) and D_max50_ (5.9 ± 0.1 mm, and 5.6 ± 0.1 mm for pine and mesquite biochars, respectively). Miscanthus biochar had the largest median grain sizes with D_min50_ of 2.7 ± 0.1 mm and D_max50_ of 10.4 ± 0.2 mm. Sewage waste biochar had the smallest D_max50_ (3.0 ± 0.0 mm) and its D_min50_ was 2.5 ± 0.0mm (Fig 6).

**Fig 7:**
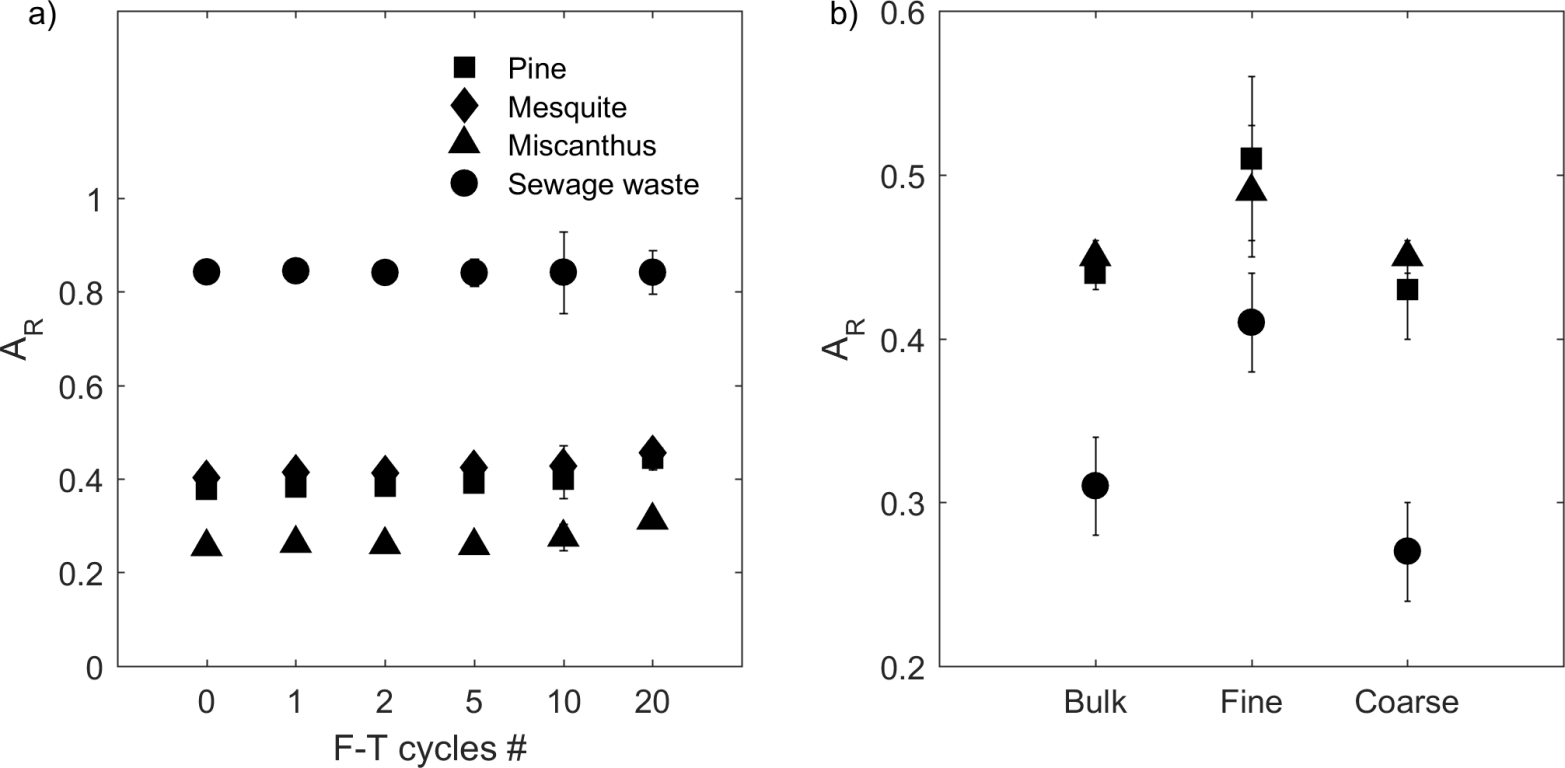
**(a) Aspect ratio (A**_**R**_ **= D**_**min50**_**/D**_**max50**_**) of all the undrained experiment biochar samples from 0 to 20 F-T cycles and (b) A**_**R**_ **of the undrained experiment pine, mesquite, and miscanthus biochars before (bulk) and after passing (fine) or being retained (coarse) by a U.S. standard #20 mesh (0.853 mm) at 20 F-T cycles.** Note: X-axis is not on a linear scale. Symbols plotted are mean values. Errors are calculated using Eq 1. Numerical data for individual replicates are presented in S2 Table.

Biochar grain shapes were visually different (Fig 1) although all feedstocks were sieved between the same mesh sizes (#8 and #6 U.S. standard mesh, 2.36–3.35 mm). Pine and mesquite biochars had a similar grain shape with pre-F-T aspect ratios (A_R_, m/m) of 0.38 ± 0.01 and 0.40 ± 0.01, respectively (Fig 7A). Pre-F-T, miscanthus biochar had the lowest A_R_ of 0.25 ± 0.01 meaning they had the most elongated shape. The highest A_R_ of 0.84 ± 0.00 was documented for sewage waste biochar indicating its more spherical shape.

The changes of grain-size distributions by F-T cycling for pine and mesquite biochars were similar with an increase in proportion of smaller particles (Figs 2A, 2B, 3A and 3B). Pine and mesquite biochars also had the same D_min50_ and D_max50_ from 1–20 F-T cycles although their pre-F-T D_min50_ and D_max50_ were slightly different. Therefore, the magnitudes of decrease in median grain size for pine and mesquite biochars from 0–20 F-T cycles were similar. From 0–20 F-T cycles, the decrease of D_min50_ was 19.9% for pine biochar and 19.6% for mesquite biochar. Similarly, the decrease of D_max50_ was 32.0% for pine biochar and 28.8% for mesquite biochar (Fig 6).

The grain-size distribution of miscanthus biochar shifted to smaller grain sizes with increasing number of F-T cycles. After 20 F-T cycles, grain-size distributions of miscanthus biochar became bimodal (Figs 2C and 3C). After 20 F-T cycles, miscanthus biochar had the largest decreases of median grain sizes with 33.9% decrease in D_min50_ and 45.8% decrease in D_max50_ (Fig 6).

We did not observe any change of grain-size distributions (Figs 2D and 3D) or median grain size (Fig 6) by F-T cycling for sewage waste biochar, likely reflecting its feedstock, which is not dominated by plant material.

The change of A_R_ with F-T cycles varied with biochar feedstock (Fig 7A) although there were no visual differences of biochar shape pre- and post- F-T cycling (Fig 1). For pine biochar, there was no significant change of A_R_ after 1 and 2 F-T cycles; however, the A_R_ was increased by 17.7% after 20 F-T cycles. The increase of A_R_ for mesquite biochar started at 2 F-T cycles with a 13.0% increase after 20 F-T cycles. The A_R_ of miscanthus biochar had a 22.4% increase after 20 F-T cycles. There was no significant change of A_R_ for sewage waste biochar (Fig 7A).

Aspect ratio also varied with biochar grain size. After 20 F-T cycles, the A_R_ of the fine biochars were higher than that of the coarse biochars for pine, mesquite, and miscanthus (Fig 7B). However, there were no significant differences of A_R_ between coarse biochar and the bulk sample (fine + coarse) for pine, mesquite, and miscanthus (Fig 7B).

### Density and intraporosity, undrained experiment

**Fig 8:**
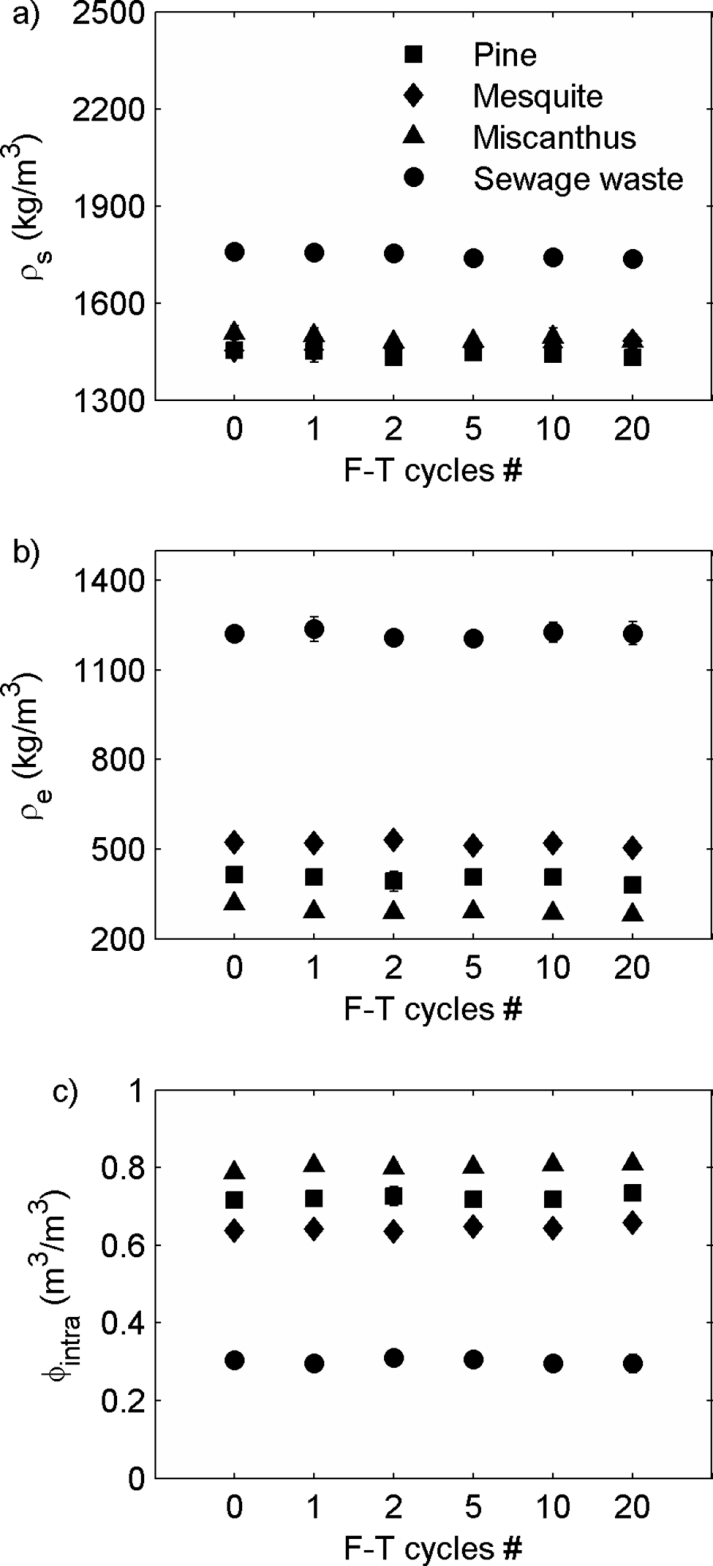
**(a) Skeletal density (ρ**_**s**_**), (b) envelope density (ρ**_**e**_**), and (c) intraporosity (ϕ**_**intra**_**) of four types of biochar from 0–20 F-T cycles in the undrained experiment.** For ρ_s_ and ρ_e_, values and errors are mean and standard deviation of triplicate samples. For ϕ_intra_, values are mean, and errors are calculated using Eq 4. Note: X-axis is not on a linear scale.

Pre-F-T, biochar’s skeletal density (ρ_s_), envelope density (ρ_e_), and intraporosity (ϕ_intra_) varied with biochar feedstock (Table 3 and Fig 8). The skeletal densities of pine, mesquite, and miscanthus biochars (1450 ± 10 kg/m^3^, 1450 ± 20 kg/m^3^, and 1510 ± 20 kg/m^3^, respectively) were statistically the same. Miscanthus biochar had the lowest ρ_e_ (320 ± 10 kg/m^3^) and the highest ϕ_intra_ (0.79 ± 0.00 m^3^/m^3^). The envelope densities of pine and mesquite biochars were 410 ± 00 kg/m^3^ and 520 ± 10 kg/m^3^, respectively, which were higher than that of miscanthus biochar. Sewage waste biochar had the highest ρ_s_ (1760 ± 00 kg/m^3^) and the highest ρ_e_ (1220 ± 10 kg/m^3^) resulting in the lowest intraporosity of 0.31 ± 0.01 m^3^/m^3^ (Table 3 and Fig 8).

**Table 3:** Skeletal density (ρ_s_, kg/m^3^), envelope density (ρ_e_, kg/m^3^), and intraporosity (ϕ_intra_, m^3^/m^3^, volume of pores/envelope particle volume) pre- and post- F-T cycles in the undrained experiment. For ρ_s_ and ρ_e_, values and errors are mean and standard deviation of triplicate samples. For ϕ_intra_, values are mean, and errors are calculated using Eq 4.

Biochar	Property	F-T cycles					
		0	1	2	5	10	20
Pine	ρ_s_ (kg/m^3^)	1450 ± 10	1450 ± 30	1430 ± 00	1450 ± 10	1440 ± 00	1430 ± 10
	ρ_e_ (kg/m^3^)	410 ± 00	410 ± 20	390 ± 30	410 ± 10	410 ± 10	380 ± 10
	ϕ_intra_ (m^3^/m^3^)	0.72 ± 0.0	0.72 ± 0.03	0.73 ± 0.06	0.72 ± 0.01	0.72 ± 0.02	0.73 ± 0.03
Mesquite	ρ_s_ (kg/m^3^)	1450 ± 20	1460 ± 10	1460 ± 10	1460 ± 00	1460 ± 10	1480 ± 00
	ρ_e_ (kg/m^3^)	520 ± 10	520 ± 10	530 ± 20	510 ± 10	520 ± 10	510 ± 00
	ϕ_intra_ (m^3^/m^3^)	0.64 ± 0.01	0.64 ± 0.02	0.64 ± 0.03	0.65 ± 0.02	0.65 ± 0.02	0.66 ± 0.02
Miscanthus	ρ_s_ (kg/m^3^)	1510 ± 20	1500 ± 20	1480 ± 10	1480 ± 10	1500 ± 30	1480 ± 00
	ρ_e_ (kg/m^3^)	320 ± 10	290 ± 20	290 ± 20	290 ± 10	290 ± 10	280 ± 10
	ϕ_intra_ (m^3^/m^3^)	0.79 ± 0.02	0.81 ± 0.05	0.80 ± 0.05	0.80 ± 0.03	0.81 ± 0.03	0.81 ± 0.04
Sewage waste	ρ_s_ (kg/m^3^)	1760 ± 00	1760 ± 00	1750 ± 00	1740 ± 10	1740 ± 10	1740 ± 00
	ρ_e_ (kg/m^3^)	1220 ± 10	1240 ± 40	1210 ± 30	1200 ± 20	1230 ± 30	1220 ± 40
	ϕ_intra_ (m^3^/m^3^)	0.31 ± 0.0	0.30 ± 0.01	0.31 ± 0.01	0.31 ± 0.01	0.30 ± 0.01	0.30 ± 0.02

The effects of F-T cycling on ρ_s_, ρ_e_, and intraporosity (ϕ_intra_) were small. There was no change of ρ_s_ for pine, mesquite, or miscanthus biochars after 20 F-T cycles. The skeletal density of sewage waste biochar decreased with F-T cycles, but the decreases were <1.3% after 20 F-T cycles (Fig 8A). There were no significant changes of ρ_e_ from 0–10 F-T cycles for pine and mesquite biochars. However, the envelope densities of these two biochars decreased by 7.9% and 3.7% after 20 F-T cycles. The envelope density of miscanthus decreased from 2–20 F-T cycles with a decrease of 12.2% after 20 F-T cycles. We did not observe any change in ρ_e_ for sewage waste biochar (Fig 8B).

The intraporosity of miscanthus biochar increased with increasing number of F-T cycles reaching a peak change of 3.2% at 20 F-T cycles. The intraporosity of pine biochar and mesquite biochar did not change until 20 F-T cycles where ϕ_intra_ of pine biochar increased by 2.5% and ϕ_intra_ of mesquite biochar increased by 3.1%. These small shifts suggest the possibility of expansion of intrapores prior to physical breakdown. There were no significant changes in ϕ_intra_ for sewage waste biochar (Fig 8C).

### Grain size, drained experiment

**Fig 9:**
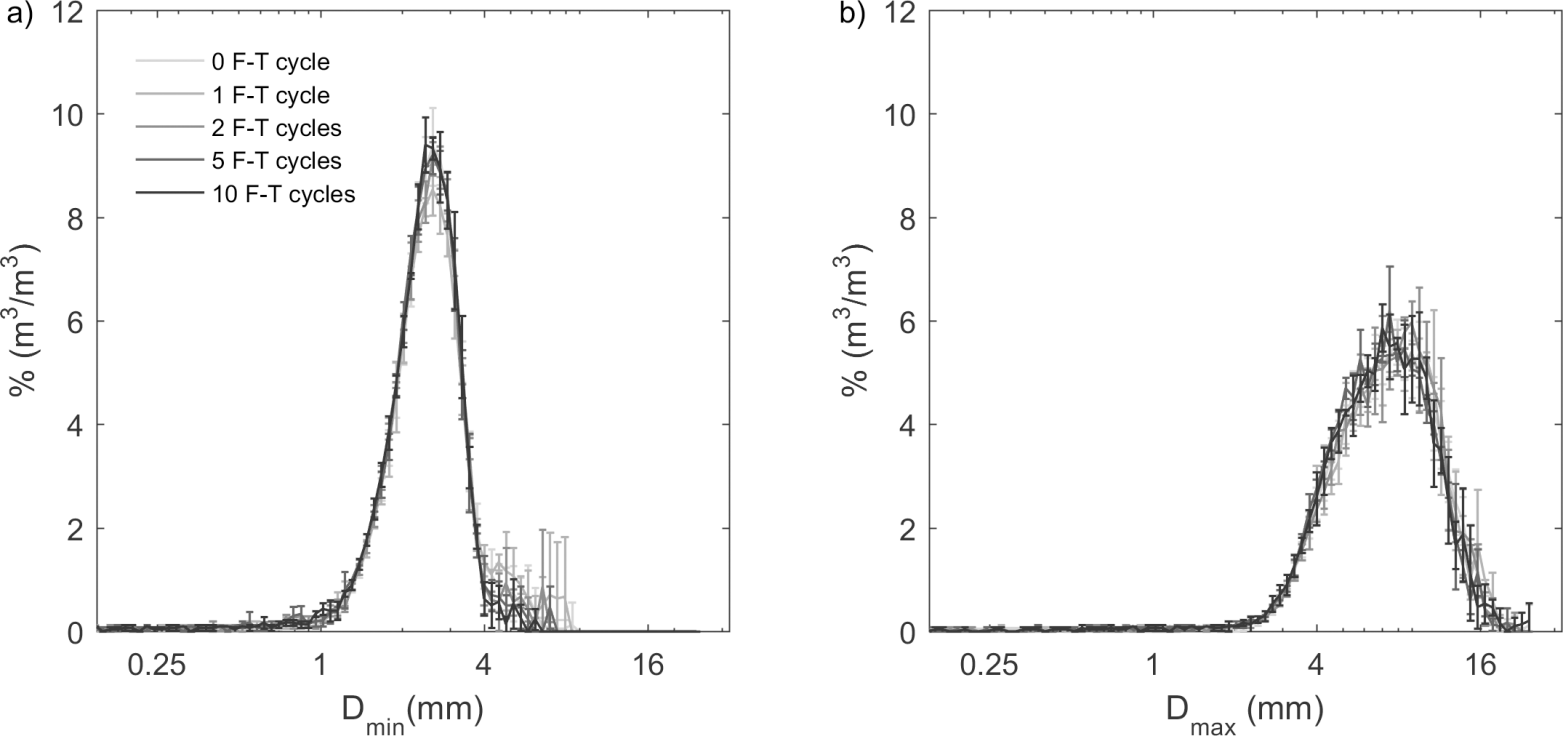
**Grain size (a) at the shortest chord (D**_**min**_**) and (b) at the maximum diameter (D**_**max**_**) distribution by volume of drained experiment mesquite biochars from 0 to 10 F-T cycles.** Values and errors are mean and standard deviation of triplicate samples. Numerical data for individual replicates are presented in S2 Table.

**Fig 10:**
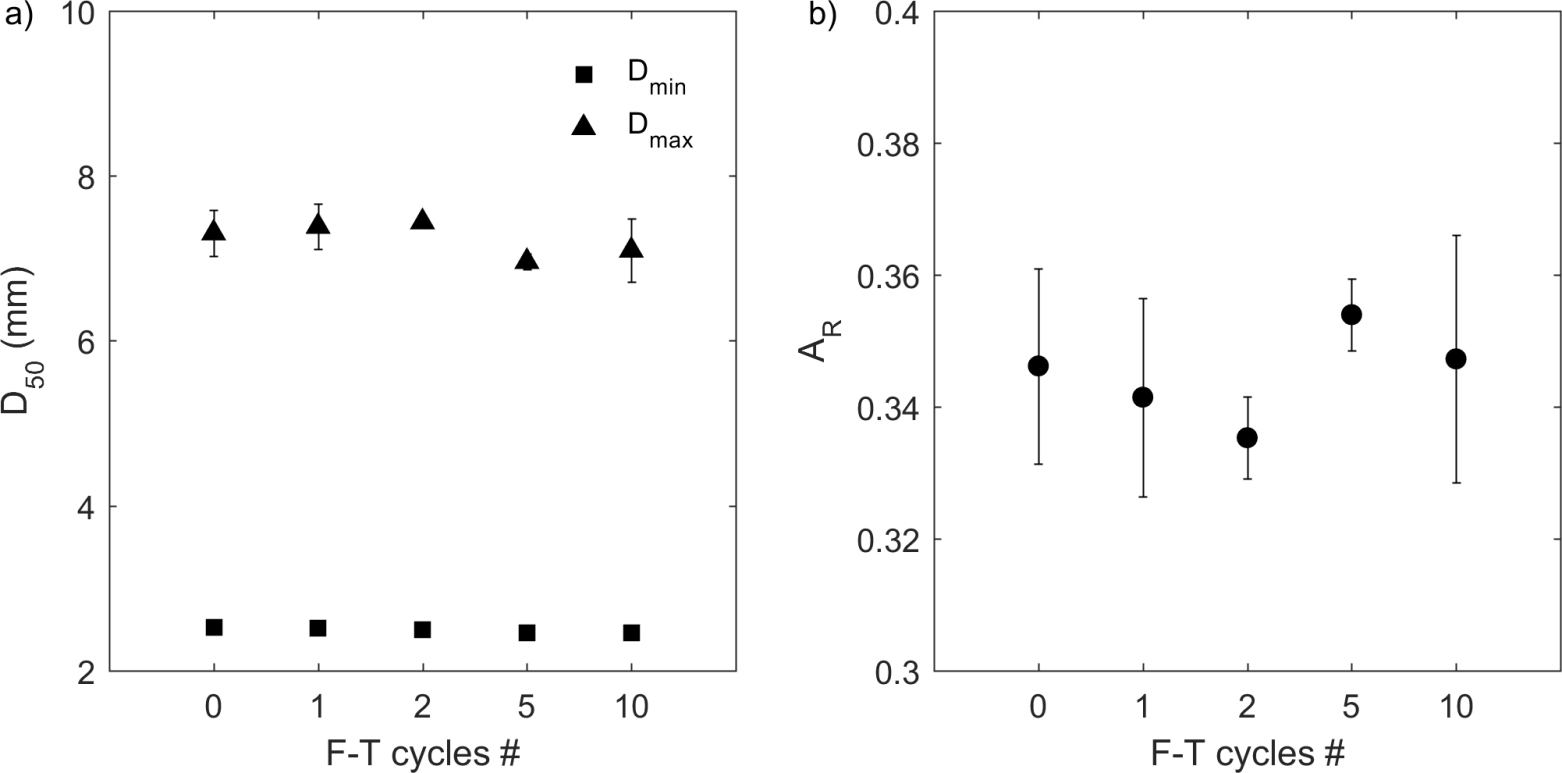
**(a) Median grain size (D**_**min50**_ **and D**_**max50**_**) and (b) aspect ratio (A**_**R**_**) of drained experiment mesquite biochars from 0–10 F-T cycles.** Values and errors are mean and standard deviation of triplicate samples. Numerical data for individual replicates are presented in S2 Table. Note: X-axis is not on a linear scale.

The changes of grain size in the drained experiment were much smaller than the undrained experiment. In the drained experiment, grain-size distributions (D_min_ and D_max_) of mesquite biochar at 0, 1, 2, 5, and 10 F-T cycles were similar (Fig 9). Median grain sizes were statistically the same before and after each F-T cycle for D_area50_ and D_max50_, however, D_min50_ showed a decrease of 4.2% at 10 F-T cycles. There was no change of A_R_ for mesquite biochar at this water content of 1.9 ± 0.3 kg/kg (Fig 10).

## Discussion

**Fig 11:**
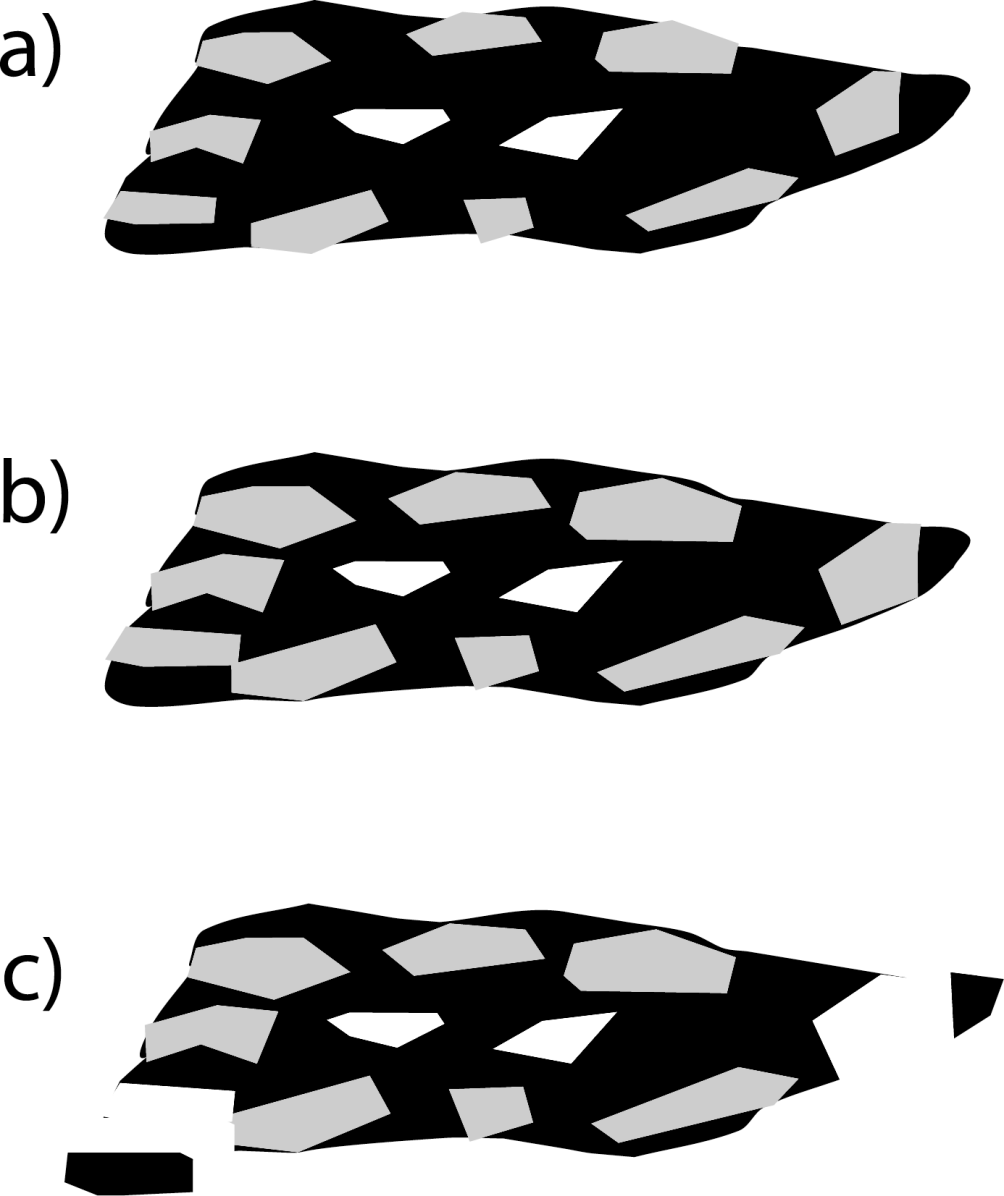
Conceptual model of how the grain size of pine, mesquite, and miscanthus biochars with higher water content was reduced by F-T cycles through loss of thin layers from the particle surface. (a) a biochar particle with its skeleton (black), connected intrapores filled with water (gray) and isolated, air-filled intrapores (white); (b) water-filled intrapores expand during freezing which increases intraporosity; and (c) biochar grain size decreases through loss of fine particles from surface of larger particles due to intrapores’ expansion.

In the undrained experiment, we observed: (1) decreases in median grain size; (2) shifts in grain-size distribution from unimodal to bimodal; and (3) increases in aspect ratio after F-T cycling for pine, mesquite, and miscanthus biochars. As these biochars physically weathered into finer particles by F-T cycling, their aspect ratio increased, meaning that they became less elongated. We also observed shifts in grain-size distribution from unimodal with only one peak to bimodal with a major peak and a minor peak. This indicates that before F-T cycles, most biochar particles had similar grain sizes (one major peak) and after F-T cycles, the finer particles produced by F-T cycles created a minor peak. By examining the grain sizes at the two peaks in each grain-size distribution curve, we found that grain size at the major peak was approximately four times larger than the grain size at the minor peak (Figs 2 and 3). We interpret that the decrease of grain size is caused by loss of thin layers on the surface (Fig 11), instead of breakage through the center of the particle. In our experiment, coarser biochar particles still dominated the bulk sample from 0–20 F-T cycles. If more F-T cycles are applied, we predict that more fine biochar particles would be produced from the surface of larger particles resulting in a larger increase in particle aspect ratio, and potentially to a shift in the major peak of grain size distribution.

In addition, we observed slight increases (up to 3.2%) of biochar intraporosity but no change of skeletal density by F-T cycling for pine, mesquite, and miscanthus biochars. However, there are documented increases of skeletal density when grinding biochar into smaller particle sizes [18]. The differences we observed indicate that F-T cycling breaks biochar particles differently compared to grinding. Liu et al. [12] suggested that the increase of skeletal density was due to breaking of isolated pores by grinding. In this study, we propose that ice crystal growth during freezing could increase biochar intrapore size due to expansion of pores that have been infiltrated by water before finally breaking biochar particles (Fig 11B).

**Fig 12:**
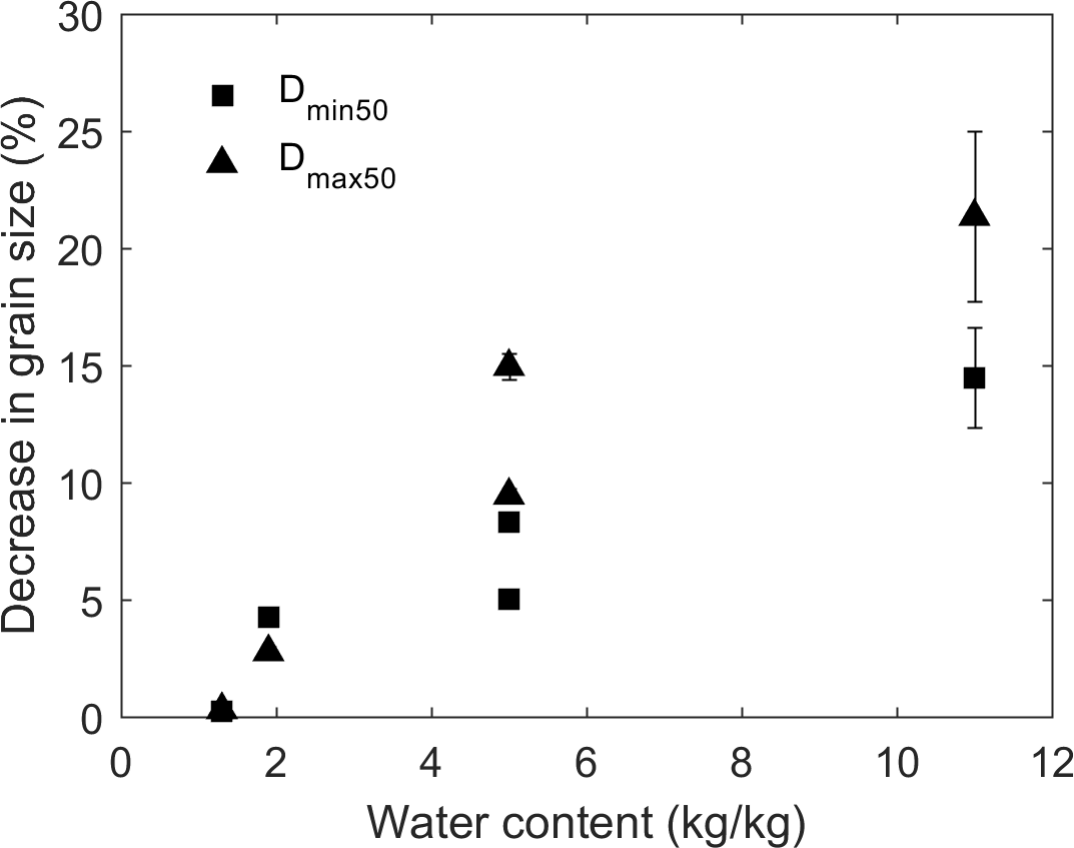
Positive Pearson correlation coefficients (R = 0.90) between decrease in median grain size and water content at 10 F-T cycles. Values and errors are mean and standard deviation of triplicate samples. Numerical data for individual replicates are presented in S2 Table.

In our experiments, biochar with water content less than 1.3 ± 0.5 kg/kg had no reductions in grain size due to F-T cycling. There is a positive correlation (R = 0.90 for all samples) between decrease of grain size and water content (Fig 12). Similarly, the experimental results of Bullock et al. [25] showed that the aggregate size of silt loam and loam were decreased by F-T cycling as water content increased when water content was 20 kg/kg. They interpreted these results as showing that forces reducing soil aggregate size were probably due to ice crystals expanding in pores, breaking particle-to-particle bonds, and splitting the soil aggregates into smaller aggregates. At lower water contents, fewer biochar particles were broken down indicating that the ice crystals completed their growth in the pores before they could apply significant force on particle bonds or biochar skeleton materials [25]. In our experiments, lower water contents were either caused by low intraporosity of biochar (i.e. sewage waste biochar in undrained experiment) or gravity drainage (i.e. mesquite biochar in drained experiment). Biochar’s intraporosity is related to biochar feedstock, pyrolysis temperature, and residence time [18]. Because biochar intraporosity and size can be controlled to some extent before application, and gravity drainage dominates in many settings, biochar can be optimally designed for different soil amendment purposes.

In summary, there are several conditions that speed up physical degradation of biochar by F-T cycling: high intraporosity and pore connectivity, large intrapores and hydrophilicity that enhances water penetration into intrapores, undrained conditions to provide enough water, and frequent F-T cycles to break the intrapores.

Our data show a decrease of grain size and an increase of aspect ratio by F-T cycling for pine, mesquite, and miscanthus biochars. From these observations, we conclude that changes will also occur in soil hydraulic conductivity and soil water retention after biochar addition to soil and exposure to F-T cycling in nature. Liu et al. [12] documented that by adding finer biochar into sand, the hydraulic conductivity decreased due to finer biochar particles filling pores between sand particles, which reduced pore throat size and increased tortuosity. Similarly, the decrease of biochar grain size by F-T cycling could decrease hydraulic conductivity in biochar-soil mixtures as the F-T-cycles produced finer particles that could migrate into pore spaces, thus increasing tortuosity and decreasing pore throat radii.

A change in biochar grain size, grain shape, and intraporosity by F-T cycling will affect soil water retention which could drive changes in plant available water. Biochar grain size and grain shape will influence the particle packing in the biochar-soil system and could change interpore volume which will impact soil water retention [14]. Biochar with lower aspect ratios (more elongated) has been found to increase water retention in the wet range (i.e. above field capacity) [14]. As aspect ratio increases and grain size decreases with F-T cycling for pine, mesquite, and miscanthus biochars, we expect water content to increase at higher (less negative) soil water potentials which will equate to more water retained by the biochar-soil mixture. Meanwhile, Liu et al. [14] found decrease of plant available water of biochar-sand mixtures after grinding biochar into smaller sizes. Liu et al. [14] also showed that plant available water in biochar-sand mixtures is mainly controlled by biochar intraporosity. However, in this study, we observed slight increases of biochar intraporosity (up to 3.2%) which may cause a small increase in plant available water.

The number of annual F-T cycles varies with region, therefore local climate will play a very important role in the effect of F-T cycling on biochar grain size. For example in the United States from the coast (proximity to Pacific Ocean, Atlantic Ocean and Gulf of Mexico) to the western Rocky Mountains, the number of F-T cycles increases from 0 to above 180 days per year [26]. Our results have shown that 20 F-T cycles can decrease biochar grain size by up to 45.8%. According to Haley [26], most regions in United States have more than 20 F-T cycles each year. As a result, we could expect a significant decrease of biochar grain size in a few years, especially for biochar amendments in the mountainous regions with sharp daily temperature changes, assuming water contents are high.

In addition, there is a feedback between the decrease of biochar grain size by F-T cycling and the increase of water retention by biochar addition affecting the longevity of biochar in soil. In these experiments we observed a positive correlation between the degree of decrease in biochar grain size and the increase of water content. In a previous study [12], we found that biochar addition could increase water retention which may have a positive impact on the decrease of biochar grain size right after biochar application in the cold regions with frequent F-T cycles. However, as biochar grain size decreases with destruction of intrapores, water retention will decrease [14], and thus the decrease in biochar grain size by F-T cycling may slow down.

## Conclusion

We investigated how F-T cycling alters grain size of four types of biochar (pine, mesquite, miscanthus, and sewage waste) prepared at two drainage conditions: an undrained experiment for all biochars and a drained experiment for mesquite biochar only.

The effect of F-T cycling on biochar grain size varied with feedstock type and drainage condition. In the undrained experiment, the grain-size distribution of miscanthus biochar shifted from unimodal to bimodal after 20 F-T cycles. The median grain size of pine, mesquite, and miscanthus biochars decreased with increasing number of F-T cycles with decreases of up to 45.8% for miscanthus biochar after 20 F-T cycles. The aspect ratio of pine, mesquite, and miscanthus biochars also increased, up to 22.4% for miscanthus biochar after 20 F-T cycles. The increase of aspect ratio means F-T cycling produced particles that were less elongated. We observed no statistically significant change in median grain size and grain shape for sewage waste biochar. The changes in skeletal density (up to 1.3% decrease), envelope density (up to 12.2% increase), and intraporosity (up to 3.2% increase) for these biochars were small. In the drained experiment, median grain size of mesquite biochar decreased by 4.2% after 10 F-T cycles.

From our laboratory experiments and analyses, we developed a conceptual model where F-T cycling decreases biochar grain size through loss of thin layers on the particle surface. These results suggest that grinding particles in the laboratory will not accurately represent how biochar will weather physically in the field. Since the effect of F-T cycling is correlated with biochar water content, biochar is more likely to break down during F-T cycling when it is wet. Furthermore, we expect the decrease of grain size and increase of aspect ratio by F-T cycling for pine, mesquite, and miscanthus biochars would drive changes in properties like soil water retention and hydraulic conductivity which are influenced by grain size and intraporosity of biochar.

## Supporting information

S1 Table Chemical composition (mg/L) of average U.S. inland rain water and synthetic rainwater used in this study. (XLS)

S2 Table Numerical data for individual replicate presented in figures in this study. Each spreadsheet contains data for each figure.
(XLS)

S1 Text Determination of Freeze and Thaw periods. (DOCX)

S1 Fig **Volumetric distribution of mesquite biochar grain size (a) at the shortest chord (D**_**min**_**); and (b) at the maximum diameter (D**_**max**_**) of a biochar grain projection before (0 day) and after being freeze for different durations (0.33, 2, 5, 10, and 20 days).** Values and errors are mean and standard deviation of triplicate samples.
(TIF)

S2 Fig **(a) median grain size (D**_**50**_**) and (b) aspect ratio (A**_**R**_**) of mesquite biochars which were freeze for different durations (8 hrs, 2, 5, 10, and 20 days) after sitting in a water bath without drainage.** The three types of median grain size (D_min50_ and D_max50_) and A_R_ were statistically the same between different durations (*p*>0.11). Values and errors are mean and standard deviation of triplicate samples.
(TIF)

S3 Fig Temperatures of room, freezer, mesquite biochar, miscanthus biochar, and sewage waste biochar during one representative freeze and thaw cycle showing that 8 hrs is enough for biochar to reach the target freezing temperature and 16 hrs is sufficient to thaw samples. (TIF)

S4 Fig Grain size at the shortest chord (D_min_), and grain size at the maximum diameter (D_max_) of a biochar grain projection. We measured grain-size distribution of biochars including D_min_ and D_max_ pre- and post- F-T cycles. From the grain-size distribution, we determined the median grain size (D_min50_, and D_max50_) and calculated the aspect ratio (A_R_ = D_min50_ / D_max50_).
(TIF)
