# Supplementary material for: Effect of freeze-thaw cycling on grain size of biochar
Source: PLoS One. 2018 Jan 12;13(1):e0191246. doi: 10.1371/journal.pone.0191246 (PMC5766239; doi:10.1371/journal.pone.0191246)
Supplement: S1 Text — (DOCX) [file pone.0191246.s003.docx]

**S1 Text. Determination of Freeze and Thaw periods.** We designed our F-T process based on the results from our trial experiment. In this experiment, we freeze mesquite biochar for 8 hrs, 2, 5, 10, and 20 days for 1 F-T cycle and measured the grain size pre and post F-T. The result showed that the median grain sizes were statistically the same after freeze mesquite biochar for 8 hrs, 2, 5, 10, and 20 days (S1 and S2 Figs). Meanwhile, the sample temperature during a F-T cycle (S3 Fig) showed that 8-hrs is enough for biochar to reach the target freezing temperature and16-hrs is sufficient to thaw samples. In addition, eight hrs plus 16 hrs is one day per F-T cycle which is convenient for scheduling experiment. Therefore, we selected 8 hrs-freeze and 16 hrs-thawing.
