# Supplementary figures and images for: Effect of freeze-thaw cycling on grain size of biochar
Source: PLoS One. 2018 Jan 12;13(1):e0191246. doi: 10.1371/journal.pone.0191246 (PMC5766239; doi:10.1371/journal.pone.0191246)

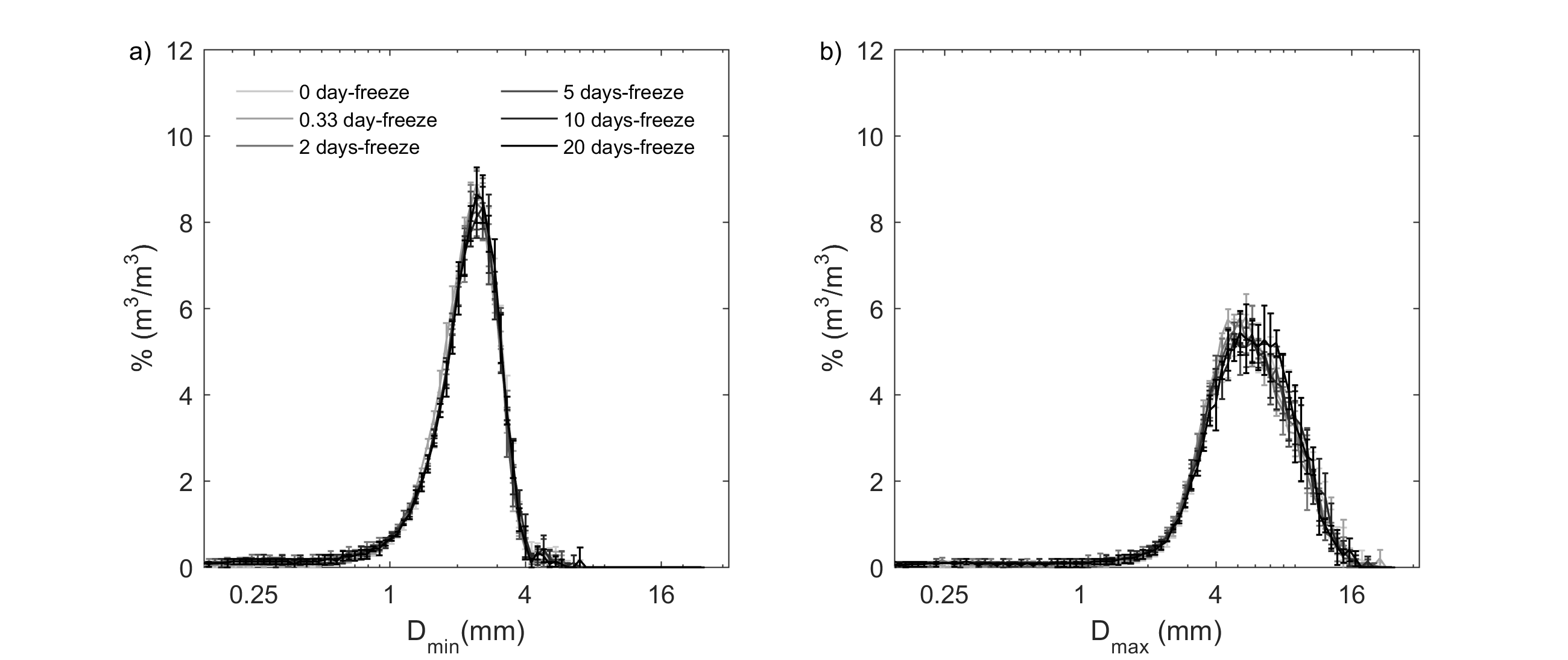

Supplement: S1 Fig — Volumetric distribution of mesquite biochar grain size (a) at the shortest chord (Dmin); and (b) at the maximum diameter (Dmax) of a biochar grain projection before (0 day) and after being freeze for different durations (0.33, 2, 5, 10, and 20 days). Values and errors are mean and standard deviation of triplicate samples. (TIF) [file pone.0191246.s004.tif]

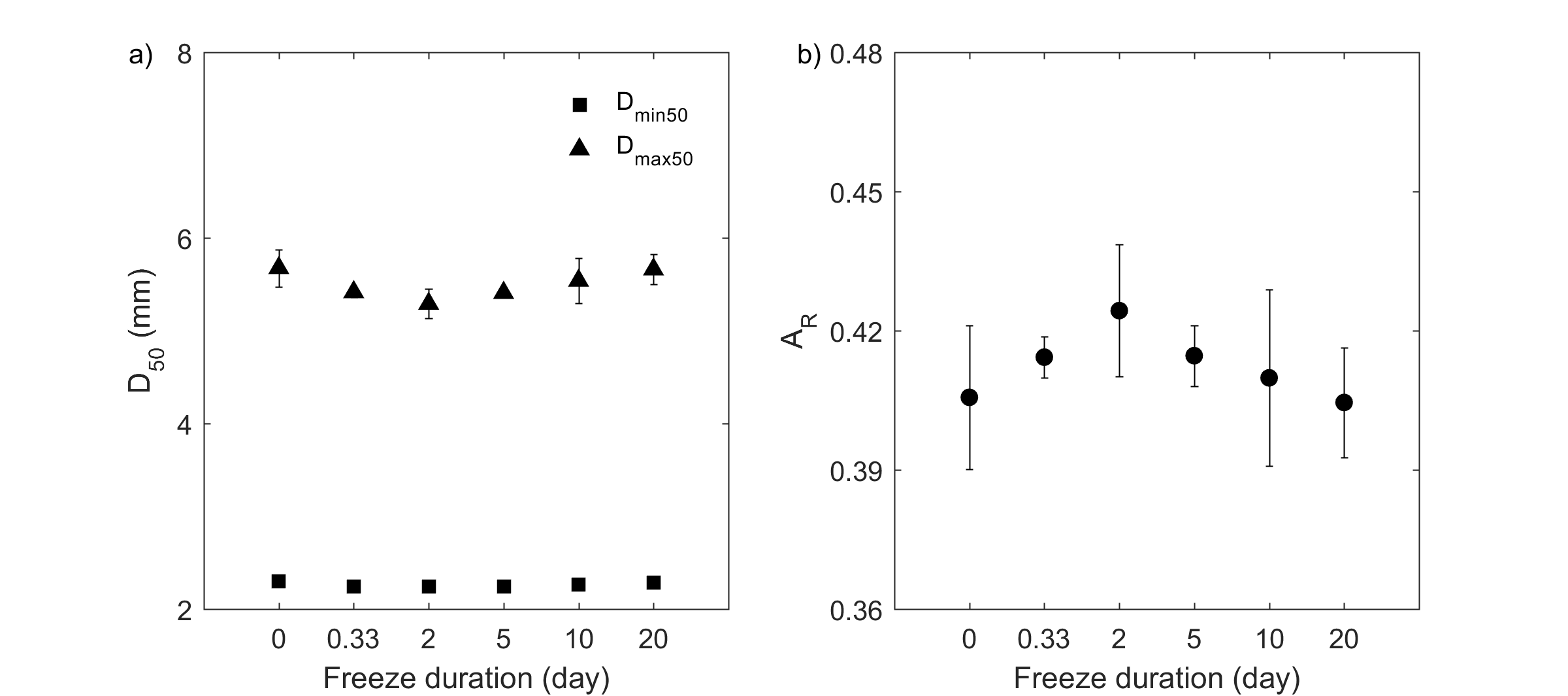

Supplement: S2 Fig — (a) median grain size (D50) and (b) aspect ratio (AR) of mesquite biochars which were freeze for different durations (8 hrs, 2, 5, 10, and 20 days) after sitting in a water bath without drainage. The three types of median grain size (Dmin50 and Dmax50) and AR were statistically the same between different durations (p>0.11). Values and errors are mean and standard deviation of triplicate samples. (TIF) [file pone.0191246.s005.tif]

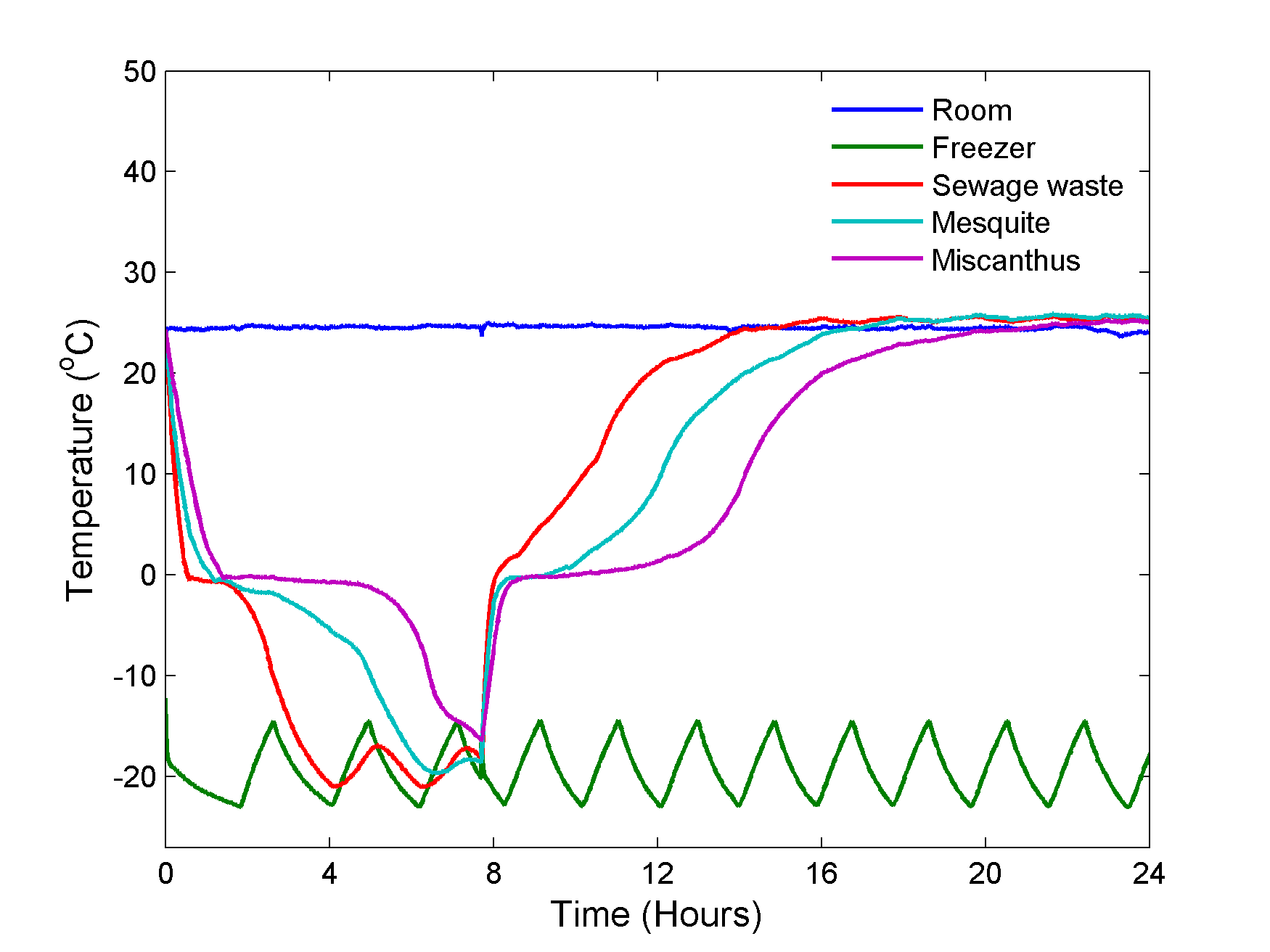

Supplement: S3 Fig — (TIF) [file pone.0191246.s006.tif]

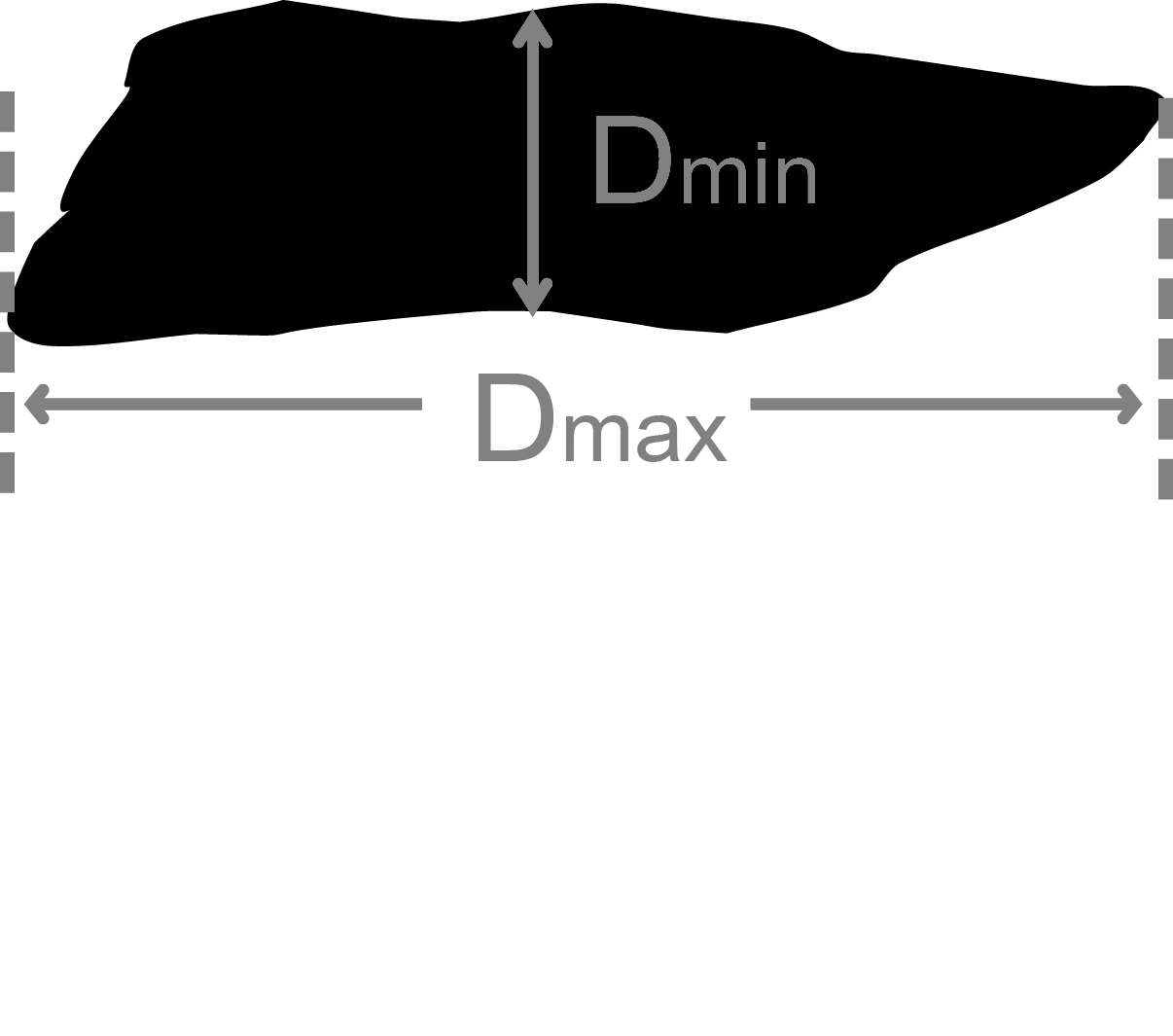

Supplement: S4 Fig — We measured grain-size distribution of biochars including Dmin and Dmax pre- and post- F-T cycles. From the grain-size distribution, we determined the median grain size (Dmin50, and Dmax50) and calculated the aspect ratio (AR = Dmin50 / Dmax50). (TIF) [file pone.0191246.s007.tif]
